# 0888. Administration of tetrahydrobiopterin (BH4) protects renal microcirculation after ischemia and reperfusion

**DOI:** 10.1186/2197-425X-2-S1-O17

**Published:** 2014-09-26

**Authors:** L Rahmania, F Su, D Orbegozo Cortés, EH Post, C Santacruz, FS Taccone, J-L Vincent, D De Backer

**Affiliations:** Erasme Hospital, Université Libre de Bruxelles, Department of Intensive Care, Brussels, Belgium

## Introduction

Abdominal aortic aneurysm surgery with supra-renal clamping is associated with potential development of renal insufficiency. Ischemia and reperfusion (I-R) produced during the procedure induces endothelial dysfunction with a decrease in tetrahydrobiopterin (BH4), a cofactor used in nitric oxide synthesis.

## Objectives

To assess whether BH4 administration could prevent the injury to the renal microcirculation caused by supra-renal aortic clamping with I-R.

## Methods

Nineteen adult sheep were anesthetized, mechanically ventilated and invasively monitored. Renal blood-flow was measured continuously through a left lumbotomy using a peri-vascular flow probe (Transonic, USA) and an aortic clamp was positioned above the renal arteries. After surgical preparation and stabilization, animals were randomized into 3 groups (SHAM=5, I-R=7, I-R+BH4=7). SHAM animals underwent surgical preparation but no aortic clamping was performed. The I-R groups were exposed to 1 hour of aortic ischemia. The I-R+BH4 group received 20 mg/kg of BH4 before aortic clamping. Animals were followed for a maximum of 6 hours after reperfusion. Renal microcirculation was evaluated at baseline, and 1, 4 and 6 hours after reperfusion using Sidestream Dark Field video-microscopy (Microvision Medical, Netherlands). We calculated perfused small vessel density (PVD), proportion of perfused small vessels (PPV) and heterogeneity of PPV (PPV-HI). Data were analyzed using the generalized estimating equation (GEE) and p-values less than 5% were considered statistically significant. Results are presented as median [IQRs].

## Results

The systemic hemodynamics variables were preserved in all 3 groups. BH4 was associated with improved renal function, as evaluated by creatinine after 6 hours of reperfusion in the I-R+BH4 group (14 mg/L [13-17]) compared to the I-R group (16 mg/L [16-22]) (P=0.072).

**Table 1:** Microcirculatory parameters in the 3 groups

	Group	Baseline	1h	4h	6h	P Group*Time
PVD (vessels/mm)	SHAM	2.6 (2.5-2.7)	2.7 (2.3-2.9)	2.4 (2.2-2.7)	2.3 (2.3-2.5) c	P<0.05
	I-R	3.2 (2.9-3.7)	2.0 (1.5-2.8) c	2.1 (1.7-2.7) c	1.6 (1.3-2.3) a, c	
	I-R+BH4	3.2 (2.7-3.4)	3.0 (2.6-3.3) b	2.5 (2.0-3.1) b, c	3.1 (2.4-3.8) b	
PPV (%)	SHAM	93.7 (93.3-94.7)	87.6 (84.2-90.6) c	83.0 (80.3-90.2) c	85.5 (82.0-90.4) c	P=0.269
	I-R	97.3 (96.0-97.9)	80.1 (73.5-90.2) c	82.7 (68.5-88.7) c	79.8 (74.9-84.6) c	
	I-R+BH4	97.8 (96.8-98.3)	92.3 (89.8-93.8) b, c	85.9 (80.9-91.5) c	89.3 (86.0-99.4) c	
PPV-HI (%)	SHAM	11.9 (8.9-13.2)	18.4 (16.5-22.8) c	14.2 (11.0-15.1)	14.4 (11.3-39.0)	P<0.05
	I-R	9.6 (5.5-11.0)	43.0 (22.2-84.0) a, c	41.8 (28.2-97.7) a, c	47.3 (11.1-76.3) c	
	I-R+BH4	9.3 (7.3-12.5)	24.7 (17.1-32.8) c	42.8 (29.1-56.2) c	25.4 (2.9-40.5) c	

*[values are presented as median with P25-75]*

a: significant difference between groups I-R and SHAM

b: significant difference between groups I-R and BH4+I-R

c: significant difference with the baseline.

## Conclusions

In a sheep model of renal I-R, BH4 pre-treatment can prevent microvascular injury and dysfunction. Clinical trials are warranted to evaluate the administration of BH4 to prevent I-R-induced kidney injury.

